# Supplementary material for: Olanzapine-induced metabolic syndrome is partially mediated by oxytocinergic system dysfunction in female Sprague-Dawley rats
Source: PLoS One. 2025 Oct 29;20(10):e0334966. doi: 10.1371/journal.pone.0334966 (PMC12571257; doi:10.1371/journal.pone.0334966)
Supplement: S1 File — (PDF) [file pone.0334966.s001.pdf]

Oral glucose tolerance test day 84					
**Time**	**Normal**	**Low dose OLZ**	**Negative control**	**Test group**	**Positive control**
0	5.2125	5.225	5.8875	5.3625	5.3
30	5.8625	5.9625	6.775	6.175	5.925
60	6.075	6.1625	7.75	6.275	6.3125
90	5.7375	5.975	7.4125	6	6
120	5.125	5.1625	6.825	5.175	5.1625
