# Supplementary material for: Olanzapine-induced metabolic syndrome is partially mediated by oxytocinergic system dysfunction in female Sprague-Dawley rats
Source: PLoS One. 2025 Oct 29;20(10):e0334966. doi: 10.1371/journal.pone.0334966 (PMC12571257; doi:10.1371/journal.pone.0334966)
Supplement: S2 File — (PDF) [file pone.0334966.s002.pdf]

Mean food intake during the induction phase

Groups	Normal	Low Dose OLZ	Negative control	Test group	Positive control
**Week 1**	149.7	150.3	146.7	152.3	149.6
**Week 2**	145.3	152.6	147.3	146.9	150.7
**Week 3**	148.1	150.7	170.7	172.3	169.6
**Week 4**	150.4	154.1	177.1	175.4	176.9
**Week 5**	149.6	151.7	179	180.1	177.6
**Week 6**	148.1	149.9	178.6	179.6	180.4
