# Supplementary material for: Olanzapine-induced metabolic syndrome is partially mediated by oxytocinergic system dysfunction in female Sprague-Dawley rats
Source: PLoS One. 2025 Oct 29;20(10):e0334966. doi: 10.1371/journal.pone.0334966 (PMC12571257; doi:10.1371/journal.pone.0334966)
Supplement: S3 File — (PDF) [file pone.0334966.s003.pdf]

Mean body weight during the induction phase

Groups	Normal	Low dose OLZ	High Dose OLZ A	High Dose OLZ B	High Dose OLZ C
**Baseline**	152.4	152.2	150.2	154.8	156.6
**Week 1**	161.2	163.4	162.6	165.2	165.6
**Week 2**	171.2	174	175	176.8	178.8
**Week 3**	181.8	186.2	187.4	187.4	189.2
**Week 4**	194.8	194.6	202.2	203.6	205.6
**Week 5**	207.2	207.6	218.2	220.2	224.8
**Week 6**	220.6	221.6	235.8	239.8	241.4
