# Supplementary material for: Olanzapine-induced metabolic syndrome is partially mediated by oxytocinergic system dysfunction in female Sprague-Dawley rats
Source: PLoS One. 2025 Oct 29;20(10):e0334966. doi: 10.1371/journal.pone.0334966 (PMC12571257; doi:10.1371/journal.pone.0334966)
Supplement: S4 File — (PDF) [file pone.0334966.s004.pdf]

Mean random blood glucose during the induction phase

Groups	Normal	Low dose OLZ	High dose OLZ A	High dose OLZ B	High dose OLZ C
**Baseline**	5.2	5.4	5.2	5.2	5.3
**Week 1**	5.2	5.2	5.1	5.3	5.2
**Week 2**	5.5	5.5	5.5	5.3	5.6
**Week 3**	5.1	5.2	5.1	4.8	5.2
**Week 4**	5.3	5.7	5.3	5.4	5.9
**Week 5**	4.8	5.7	6.7	7.0	6.9
**Week 6**	5.9	5.9	7.8	7.6	7.9
