# Supplementary material for: Olanzapine-induced metabolic syndrome is partially mediated by oxytocinergic system dysfunction in female Sprague-Dawley rats
Source: PLoS One. 2025 Oct 29;20(10):e0334966. doi: 10.1371/journal.pone.0334966 (PMC12571257; doi:10.1371/journal.pone.0334966)
Supplement: S5 File — (PDF) [file pone.0334966.s005.pdf]

Oral glucose tolerance test day 42					
**Time**	**Normal**	**Low dose OLZ**	**High dose OLZ A**	**High dose OLZ B**	**High dose OLZ C**
0	5.25	5.33	5.79	5.78	5.49
30	5.95	6.06	6.73	6.87	6.71
60	6.15	6.28	7.76	7.42	7.72
90	5.58	5.68	7.37	7.37	7.47
120	5.01	5.11	7.16	6.62	6.5
