# Supplementary material for: Olanzapine-induced metabolic syndrome is partially mediated by oxytocinergic system dysfunction in female Sprague-Dawley rats
Source: PLoS One. 2025 Oct 29;20(10):e0334966. doi: 10.1371/journal.pone.0334966 (PMC12571257; doi:10.1371/journal.pone.0334966)
Supplement: S6 File — (PDF) [file pone.0334966.s006.pdf]

Mean food intake during the treatment phase

Groups	Normal	Low Dose OLZ	Negative control	Test group	Positive control
**Week 7**	157.3	158.3	184.7	176.3	179.1
**Week 8**	160.1	159.9	188.6	170.3	173.7
**Week 9**	159.3	163	190.1	161.4	163.3
**Week 10**	160.9	159.9	196.3	162.4	160.4
**Week 11**	159.9	161.3	193.7	158.1	162.7
**Week 12**	160.9	163.7	196.6	162.6	164.1
