# Supplementary material for: Olanzapine-induced metabolic syndrome is partially mediated by oxytocinergic system dysfunction in female Sprague-Dawley rats
Source: PLoS One. 2025 Oct 29;20(10):e0334966. doi: 10.1371/journal.pone.0334966 (PMC12571257; doi:10.1371/journal.pone.0334966)
Supplement: S7 File — (PDF) [file pone.0334966.s007.pdf]

Mean body weight during the treatment phase

Groups	Normal	Low dose OLZ	Negative control	Test group	Positive control
**Week 7**	234.6	236.2	260.6	261	261.6
**Week 8**	245.6	248.6	281.2	274.6	272.2
**Week 9**	256.2	262	306	270	273.4
**Week 10**	268.8	274.8	336	274.8	275.4
**Week 11**	280.6	288.4	366.8	287.6	288.4
**Week 12**	287.4	297.2	376.2	297.2	299.4
