# Supplementary material for: Olanzapine-induced metabolic syndrome is partially mediated by oxytocinergic system dysfunction in female Sprague-Dawley rats
Source: PLoS One. 2025 Oct 29;20(10):e0334966. doi: 10.1371/journal.pone.0334966 (PMC12571257; doi:10.1371/journal.pone.0334966)
Supplement: S8 File — (PDF) [file pone.0334966.s008.pdf]

Mean random blood glucose during the treatment phase

Groups	Normal	Low dose OLZ	Negative control	Test group	Positive control
**Week 7**	5.2	5.3	7.8	7.7	7.7
**Week 8**	5.5	5.7	7.9	6.5	6.6
**Week 9**	5.4	5.6	8.7	5.6	5.6
**Week 10**	5.6	5.8	9.2	5.8	5.7
**Week 11**	5.7	5.8	9.6	5.8	5.9
**Week 12**	5.5	5.7	9.8	5.8	5.6
