# Supplementary material for: Olanzapine-induced metabolic syndrome is partially mediated by oxytocinergic system dysfunction in female Sprague-Dawley rats
Source: PLoS One. 2025 Oct 29;20(10):e0334966. doi: 10.1371/journal.pone.0334966 (PMC12571257; doi:10.1371/journal.pone.0334966)
Supplement: S9 File — (PDF) [file pone.0334966.s009.pdf]

Oral glucose tolerance test day 63					
**Time**	**Normal**	**Low dose OLZ**	**Negative control**	**Test group**	**Positive control**
0	5.1875	5.25	5.925	5.7375	5.65
30	5.9	6.0875	6.7375	6.65	6.575
60	6.125	6.225	7.825	7.4125	7.4875
90	5.675	5.7	7.325	7.2375	7.3
120	5	5.1875	6.8875	6.475	6.5375
