# Supplementary material for: Olanzapine-induced metabolic syndrome is partially mediated by oxytocinergic system dysfunction in female Sprague-Dawley rats
Source: PLoS One. 2025 Oct 29;20(10):e0334966. doi: 10.1371/journal.pone.0334966 (PMC12571257; doi:10.1371/journal.pone.0334966)
Supplement: S11 File — (PDF) [file pone.0334966.s011.pdf]

Liver weight

Groups	Normal	Low Dose OLZ	Negative control	Test group	Positive control
**1**	7.51	7.44	12.42	7.16	7.96
**2**	5.25	7.62	10.82	6.7	5.29
**3**	6.3	6.68	11.09	6.12	6.49
**4**	6.01	6.14	10.6	6.26	7.59
**5**	5.14	6.2	10.89	7.02	6.52
