# Supplementary material for: Olanzapine-induced metabolic syndrome is partially mediated by oxytocinergic system dysfunction in female Sprague-Dawley rats
Source: PLoS One. 2025 Oct 29;20(10):e0334966. doi: 10.1371/journal.pone.0334966 (PMC12571257; doi:10.1371/journal.pone.0334966)
Supplement: S12 File — (PDF) [file pone.0334966.s012.pdf]

Hepatic index

Groups	Normal	Low Dose OLZ	Negative control	Test group	Positive control
**1**	0.026821	0.0248	0.033477089	0.024189189	0.027448276
**2**	0.018293	0.0254	0.030055556	0.022333333	0.017692308
**3**	0.021724	0.022491582	0.028005051	0.0204	0.020408805
**4**	0.020512	0.020673401	0.027894737	0.020866667	0.027107143
**5**	0.017909	0.021232877	0.029117647	0.024206897	0.021032258
