# Supplementary material for: Olanzapine-induced metabolic syndrome is partially mediated by oxytocinergic system dysfunction in female Sprague-Dawley rats
Source: PLoS One. 2025 Oct 29;20(10):e0334966. doi: 10.1371/journal.pone.0334966 (PMC12571257; doi:10.1371/journal.pone.0334966)
Supplement: S13 File — (PDF) [file pone.0334966.s013.pdf]

Hepatic triglyceride levels

**Groups**	**Normal**	**Low Dose OLZ**	**Negative control**	**Test group**	**Positive control**
**1**	5.22	4.1	6.54	3.36	4
**2**	4.17	5.73	7.3	4.97	5.03
**3**	3.89	4.26	8.12	4.25	4.13
**4**	4.56	3.49	8.87	4.01	3.95
**5**	3.36	3.92	8.72	4.36	4.14
