# Supplementary material for: Olanzapine-induced metabolic syndrome is partially mediated by oxytocinergic system dysfunction in female Sprague-Dawley rats
Source: PLoS One. 2025 Oct 29;20(10):e0334966. doi: 10.1371/journal.pone.0334966 (PMC12571257; doi:10.1371/journal.pone.0334966)
Supplement: S14 File — (PDF) [file pone.0334966.s014.pdf]

Hepatic steatosis area

**Groups**	**Normal**	**Low Dose OLZ**	**Negative control**	**Test group**	**Positive control**
**1**	2.2	5.7	29.4	10.4	6.9
**2**	3.4	3.6	33.9	7.8	12.5
**3**	1.6	4.2	18.6	13.9	8.3
**4**	0.9	4.2	15.9	8.6	12.1
**5**	4.8	3.1	26.7	7.7	7.3
