# Supplementary material for: Olanzapine-induced metabolic syndrome is partially mediated by oxytocinergic system dysfunction in female Sprague-Dawley rats
Source: PLoS One. 2025 Oct 29;20(10):e0334966. doi: 10.1371/journal.pone.0334966 (PMC12571257; doi:10.1371/journal.pone.0334966)
Supplement: S15 File — (PDF) [file pone.0334966.s015.pdf]

Mesenteric adipose tissue weight

Groups	Normal	Low dose OLZ	Negative control	Test group	Positive control
**1**	1.89	1.87	2.8	1.36	1.73
**2**	1.9	2.94	5.03	2.12	2.57
**3**	1.97	2.24	3.07	1.75	2.01
**4**	1.91	2.35	2.98	0.98	1.46
**5**	2.01	2.86	3.37	1.81	1.95
