# Supplementary material for: Olanzapine-induced metabolic syndrome is partially mediated by oxytocinergic system dysfunction in female Sprague-Dawley rats
Source: PLoS One. 2025 Oct 29;20(10):e0334966. doi: 10.1371/journal.pone.0334966 (PMC12571257; doi:10.1371/journal.pone.0334966)
Supplement: S16 File — (PDF) [file pone.0334966.s016.pdf]

Retroperitoneal adipose tissue weight

Groups	Normal	Low dose OLZ	Negative control	Test group	Positive control
**1**	2.41	1.25	1.85	1.81	0.96
**2**	1.48	1.95	5.66	1.72	1.52
**3**	1.62	2.41	2.93	1.1	1.8
**4**	1.32	1.88	3.25	0.74	1.53
**5**	1.49	1.96	2.97	1.96	1.71
