# Supplementary material for: Olanzapine-induced metabolic syndrome is partially mediated by oxytocinergic system dysfunction in female Sprague-Dawley rats
Source: PLoS One. 2025 Oct 29;20(10):e0334966. doi: 10.1371/journal.pone.0334966 (PMC12571257; doi:10.1371/journal.pone.0334966)
Supplement: S17 File — (PDF) [file pone.0334966.s017.pdf]

Pericardial adipose tissue weight

Groups	Normal	Low dose OLZ	Negative control	Test group	Positive control
**1**	0.12	0.08	0.19	0.06	0.13
**2**	0.05	0.13	0.36	0.06	0.07
**3**	0.01	0.04	0.17	0.17	0.11
**4**	0.14	0.21	0.23	0.04	0.08
**5**	0.11	0.03	0.15	0.11	0.07
