# Supplementary material for: Olanzapine-induced metabolic syndrome is partially mediated by oxytocinergic system dysfunction in female Sprague-Dawley rats
Source: PLoS One. 2025 Oct 29;20(10):e0334966. doi: 10.1371/journal.pone.0334966 (PMC12571257; doi:10.1371/journal.pone.0334966)
Supplement: S18 File — (PDF) [file pone.0334966.s018.pdf]

**Total visceral adipose tissue weight**

| Groups | Normal | Low dose OLZ | Negative control | Test group | Positive control |
|---|---|---|---|---|---|
| **1** | 4.42 | 3.2 | 4.84 | 3.23 | 2.82 |
| **2** | 3.43 | 5.02 | 11.05 | 3.9 | 4.16 |
| **3** | 3.6 | 4.69 | 6.17 | 3.02 | 3.92 |
| **4** | 3.37 | 4.44 | 6.46 | 1.76 | 3.07 |
| **5** | 3.61 | 4.85 | 6.49 | 3.88 | 3.73 |
