# Supplementary material for: Olanzapine-induced metabolic syndrome is partially mediated by oxytocinergic system dysfunction in female Sprague-Dawley rats
Source: PLoS One. 2025 Oct 29;20(10):e0334966. doi: 10.1371/journal.pone.0334966 (PMC12571257; doi:10.1371/journal.pone.0334966)
Supplement: S19 File — (PDF) [file pone.0334966.s019.pdf]

Total serum cholesterol

Groups	Normal	Low dose OLZ	Negative control	Test group	Positive control
**1**	4.9	5.5	7.5	4.9	5.6
**2**	5.4	5.9	7.4	6.7	4.7
**3**	4.8	5.1	7.3	5.8	6.1
**4**	5.1	6.4	6.9	5.4	6.9
**5**	5.7	5.2	7.1	5.4	5.5
