# Supplementary material for: Olanzapine-induced metabolic syndrome is partially mediated by oxytocinergic system dysfunction in female Sprague-Dawley rats
Source: PLoS One. 2025 Oct 29;20(10):e0334966. doi: 10.1371/journal.pone.0334966 (PMC12571257; doi:10.1371/journal.pone.0334966)
Supplement: S20 File — (PDF) [file pone.0334966.s020.pdf]

Serum triglyceride

**Groups**	**Normal**	**Low dose OLZ**	**Negative control**	**Test group**	**Positive control**
**1**	1.7	2.7	7.4	3.2	2.9
**2**	2.5	2.8	6.8	2.7	3.6
**3**	2.7	3.1	7.2	2.5	3.1
**4**	3.2	2.9	6.4	3.4	2.8
**5**	3.9	3.7	6.1	3.7	3
