# Supplementary material for: Olanzapine-induced metabolic syndrome is partially mediated by oxytocinergic system dysfunction in female Sprague-Dawley rats
Source: PLoS One. 2025 Oct 29;20(10):e0334966. doi: 10.1371/journal.pone.0334966 (PMC12571257; doi:10.1371/journal.pone.0334966)
Supplement: S21 File — (PDF) [file pone.0334966.s021.pdf]

LDL-C

**Groups**	**Normal**	**Low dose OLZ**	**Negative control**	**Test group**	**Positive control**
**1**	1.8	2.3	5.6	2.5	1.4
**2**	2	2.1	6.7	2.8	1.8
**3**	2.1	1.7	5.9	1.8	3
**4**	1.6	2.5	6.1	2.1	2.9
**5**	2.1	1.9	4.9	2.4	2.3
