# Supplementary material for: Olanzapine-induced metabolic syndrome is partially mediated by oxytocinergic system dysfunction in female Sprague-Dawley rats
Source: PLoS One. 2025 Oct 29;20(10):e0334966. doi: 10.1371/journal.pone.0334966 (PMC12571257; doi:10.1371/journal.pone.0334966)
Supplement: S22 File — (PDF) [file pone.0334966.s022.pdf]

HDL-C

**Groups**	**Normal**	**Low dose OLZ**	**Negative control**	**Test group**	**Positive control**
**1**	1.9	1.6	0.9	1.4	1.8
**2**	1.1	2.3	1.2	1.5	1.9
**3**	2.4	1.9	0.4	1.7	2
**4**	2.2	1.8	0.7	2.3	1.7
**5**	2.5	1.4	0.9	2.2	1.7
