# Supplementary material for: Olanzapine-induced metabolic syndrome is partially mediated by oxytocinergic system dysfunction in female Sprague-Dawley rats
Source: PLoS One. 2025 Oct 29;20(10):e0334966. doi: 10.1371/journal.pone.0334966 (PMC12571257; doi:10.1371/journal.pone.0334966)
Supplement: S1 Table — (PDF) [file pone.0334966.s023.pdf]

Body weight (g) (induction phase)						
**Groups**	**Normal control**	**Low dose OLZ**	**High dose OLZ A**	**High dose OLZ B**	**High dose OLZ C**	**P value**
Week 0 (baseline)	152.4 ± 1.939	150.2 ± 2.177	152.2 ± 1.241	154.8 ± 1.594	156.6 ± 3.124	0.2748
Week 1	152.4 ± 1.939	152.2 ± 2.241	150.2 ± 2.177	154.8 ± 1.594	156.6 ± 3.124	0.2748
Week 2	171.2 ± 1.828	174.0 ± 2.608	175.0 ± 2.864	176.8 ± 2.709	178.8 ± 4.903	0.8294
Week 3	181.8 ± 1.934	186.2 ± 2.267	187.4 ± 2.943	187.4 ± 3.326	189.2 ± 4.283	0.5238
Week 4	194.8 ± 2.956	194.6 ± 1.536	202.0 ± 3.760	203.6 ± 4.697	205.6 ± 5.455	0.1880
Week 5	207.2 ± 4.443	207.6 ± 2.502	218.2 ± 2.538	220.2 ± 3.023	224.8 ± 2.332	0.0015
Week 6	220.6 ± 2.926	221.6 ± 1.631	235.8 ± 4.913	239.8 ± 2.437	241.4 ± 1.503	< 0.0001
