# Supplementary material for: Olanzapine-induced metabolic syndrome is partially mediated by oxytocinergic system dysfunction in female Sprague-Dawley rats
Source: PLoS One. 2025 Oct 29;20(10):e0334966. doi: 10.1371/journal.pone.0334966 (PMC12571257; doi:10.1371/journal.pone.0334966)
Supplement: S2 Table — (PDF) [file pone.0334966.s024.pdf]

Body weight (g) (Treatment phase)						
**Groups**	**Normal control**	**Low dose OLZ**	**Negative control**	**Test group**	**Positive control**	**P value**
Week 7	234.6 ± 3.385	236.2 ± 3.826	260.6 ± 2.159	261.0 ± 1.581	261.6 ± 1.913	< 0.0001
Week 8	245.6 ± 1.536	248.6 ± 4.308	281.2 ± 2.417	274.6 ± 2.088	272.2 ± 4.841	< 0.0001
Week 9	256.2 ± 1.158	262.0 ± 3.066	306.0 ± 3.268	270.0 ± 2.608	272.2 ± 4.841	< 0.0001
Week 10	268.8 ± 0.8602	274.8 ± 3.693	336.0 ± 4.037	274.8 ± 2.746	275.4 ± 1.435	< 0.0001
Week 11	280.6 ± 2.857	288.4 ± 1.077	366.8 ± 4.409	287.6 ± 1.327	288.4 ± 2.315	< 0.0001
Week 12	287.4 ± 2.159	297.2 ± 1.463	376.2 ± 5.919	297.2 ± 1.960	299.4 ± 6.794	< 0.0001
