# Supplementary material for: Olanzapine-induced metabolic syndrome is partially mediated by oxytocinergic system dysfunction in female Sprague-Dawley rats
Source: PLoS One. 2025 Oct 29;20(10):e0334966. doi: 10.1371/journal.pone.0334966 (PMC12571257; doi:10.1371/journal.pone.0334966)
Supplement: S3 Table — (PDF) [file pone.0334966.s025.pdf]

Lipid profile (mmol/L) (Treatment phase)						
**Groups**	**Normal control**	**Low dose OLZ**	**Negative control**	**Test group**	**Positive control**	**P value**
Total serum cholesterol	5.180 ± 0.1655	5.620 ± 0.2396	7.240 ± 0.1077	5.640 ± 0.3010	5.760 ± 0.3628	= 0.0002
Serum triglycerides	2.800 ± 0.3661	3.040 ± 0.1778	6.780 ± 0.2417	3.100 ± 0.2214	3.080 ± 0.1393	< 0.0001
LDL-c	1.920 ± 0.09695	2.100 ± 0.1414	5.840 ± 0.2960	2.320 ± 0.1715	2.280 ± 0.3098	< 0.0001
HDL-c	2.020 ± 0.2518	1.800 ± 0.1517	0.8200 ± 0.1319	1.820 ± 0.1828	1.820 ± 0.05831	= 0.0005
