# Supplementary material for: Olanzapine-induced metabolic syndrome is partially mediated by oxytocinergic system dysfunction in female Sprague-Dawley rats
Source: PLoS One. 2025 Oct 29;20(10):e0334966. doi: 10.1371/journal.pone.0334966 (PMC12571257; doi:10.1371/journal.pone.0334966)
Supplement: Attachment — Submitted filename: Response to Reviewers.docx [file pone.0334966.s027.docx]

**To the Editor, PLoS ONE,**

The authors would like to thank the journal for the high-quality and professional peer review of our manuscript. The editorial, as well as the peer review comments, have greatly improved the quality of the manuscript. Here are our detailed responses as well as corrections to the editorial and peer review comments.

**Journal Requirements**

1. **Editor:** Please ensure that your manuscript meets PLOS ONE's style requirements, including those for file naming. The PLOS ONE style templates can be found at

<https://journals.plos.org/plosone/s/file?id=wjVg/PLOSOne_formatting_sample_main_body.pdf> and 
<https://journals.plos.org/plosone/s/file?id=ba62/PLOSOne_formatting_sample_title_authors_affiliations.pdf>

**Authors’ response:** The authors’ appreciate the editor’s comment and the manuscript has been revised to meet PLOS ONE requirements.

2. **Editor:** We note that your Data Availability Statement is currently as follows:

“All relevant data are within the manuscript and its Supporting Information files”

Please confirm at this time whether or not your submission contains all raw data required to replicate the results of your study. Authors must share the “minimal data set” for their submission. PLOS defines the minimal data set to consist of the data required to replicate all study findings reported in the article, as well as related metadata and methods (<https://journals.plos.org/plosone/s/data-availability#loc-minimal-data-set-definition>).

For example, authors should submit the following data:

- The values behind the means, standard deviations and other measures reported;
- The values used to build graphs;
- The points extracted from images for analysis.

Authors do not need to submit their entire data set if only a portion of the data was used in the reported study.

If your submission does not contain these data, please either upload them as Supporting Information files or deposit them to a stable, public repository and provide us with the relevant URLs, DOIs, or accession numbers. For a list of recommended repositories, please see <https://journals.plos.org/plosone/s/recommended-repositories>.

If there are ethical or legal restrictions on sharing a de-identified data set, please explain them in detail (e.g., data contain potentially sensitive information, data are owned by a third-party organization, etc.) and who has imposed them (e.g., an ethics committee). Please also provide contact information for a data access committee, ethics committee, or other institutional body to which data requests may be sent. If data are owned by a third party, please indicate how others may request data access.

**Authors’ response:** The authors appreciate the editor’s comment and the authors have uploaded the minimal data as Supporting information files.

3. **Editor:** PLOS requires an ORCID iD for the corresponding author in Editorial Manager on papers submitted after December 6th, 2016. Please ensure that you have an ORCID iD and that it is validated in Editorial Manager. To do this, go to ‘Update my Information’ (in the upper left-hand corner of the main menu), and click on the Fetch/Validate link next to the ORCID field. This will take you to the ORCID site and allow you to create a new iD or authenticate a pre-existing iD in Editorial Manager.

**Authors’ response:** We thank the editor for this reminder. The corresponding author’s ORCID iD has been added and successfully validated in Editorial Manager.

4. Please upload a new copy of Figures 5 to 8 as the detail is not clear. Please follow the link for more information:

<https://blogs.plos.org/plos/2019/06/looking-good-tips-for-creating-your-plos-figures-graphics/>
<https://blogs.plos.org/plos/2019/06/looking-good-tips-for-creating-your-plos-figures-graphics/>

**Authors’ response:** We thank the editor for the comment. All figures have been revised to follow PLOS ONE guidelines, and the Results section has been restructured as suggested. As a result, the manuscript now includes 13 figures, all formatted according to the journal’s requirements.

EDITOR AND REVIEWERS COMMENTS:

1. **Editor:** The title is clear but overly cautious with the phrase “appears to be partially caused.” Consider revising to a more assertive and specific title, such as: “Olanzapine-Induced Metabolic Syndrome Is Partially Mediated by Oxytocinergic System Dysfunction in Female Sprague-Dawley Rats”.

**Authors’ response:** We thank the reviewer for this comment. We have revised it as suggested.

2. **Editor:** The abstract lacks sufficient methodological details (e.g., number of rats, doses, and study duration) and specific results (e.g., effect sizes or numerical values). Revise the abstract to include these details, ensure it is concise (≤300 words), and strengthen the conclusion by linking findings to potential clinical implications or future research directions.

**Authors’ response**: The authors acknowledge the editor’s comment. We have added methodological detail: number of rats, doses and study duration, and specific results in the abstract. We have revised the conclusion in the abstract to explicitly link our findings to clinical implications and future research directions. The revised text now reads: “OLZ-induced metabolic abnormalities were mitigated by oxytocin, indicating that the oxytocinergic system hypofunction may be implicated in its pathophysiology. These results highlight OT’s therapeutic potential and call for further clinical research to explore its role in the management of antipsychotic-induced metabolic syndrome.” We believe this revision clearly conveys both the potential clinical relevance and the need for future studies.

3. **Editor:** The ethics statement is incomplete, lacking details on the approving ethics committee, approval number, and adherence to animal welfare guidelines. Please provide a comprehensive statement, including the committee name and approval number.

**Authors’ response:** We thank the editor for this observation. We would like to respectfully clarify that the ethics statement, including the approving ethics committee, approval number, and adherence to animal welfare guidelines, is currently included in the manuscript. The ethics statement in the manuscript is as follows: “Ethical approval for the experimental study was obtained from the Biosafety, Animal Use and Ethics Committee, Department of Veterinary Anatomy and Physiology, Faculty of Veterinary Medicine, University of Nairobi, and FVM BAUEC/2023/440 was the permit number issued. All surgical procedures were carried out under sodium pentobarbital anesthesia, with rigorous measures taken to reduce distress and discomfort.”

4. **Editor:** The data availability statement is vague and does not meet PLOS ONE’s open data policy. Upload data to a public repository or provide a clear justification for restricted access, including contact details for data requests (e.g., “Data are available from the University of Nairobi Institutional Data Access Committee [contact email]”).

**Authors’ response:** We thank the editor for this comment. All relevant data are included in the Supporting Information files, which are publicly accessible with the manuscript.

5. **Editor:** The results section is repetitive and lacks structure, making it difficult to follow trends across groups. Organize results into clear subsections (e.g., induction phase outcomes, treatment phase outcomes, inter-group comparisons) and include summary tables or figures to consolidate key findings (e.g., body weight, food intake, lipid profiles).

**Authors’ response:** We appreciate the editor’s insightful suggestion. We have restructured the Results section into clear subsections and added figures to consolidate key findings. We have included summary tables under Supporting Information files (S24 Table, S23 Table and S25 Table).

6. **Editor:** Ensure figures meet PLOS ONE’s technical requirements (≥300 dpi, TIFF/EPS format) and include detailed legends explaining content and statistical significance.

**Authors’ response:** We appreciate the editor’s guidance regarding figure quality and presentation. All figures have been revised to comply with PLOS ONE’s technical requirements and included detailed legends and captions explaining content and statistical significance.

7. **Editor:** The introduction is overly lengthy and lacks focus on the oxytocinergic system’s role. Streamline it into three sections: background on olanzapine and metabolic syndrome, oxytocin’s role in metabolism, and study objectives with a clear hypothesis.

**Author’s response:** We sincerely thank the editor for this valuable feedback. In line with the recommendation, we have revised and streamlined the Introduction into three focused sections as suggested. We believe this restructuring improves the clarity, flow, and focus of the Introduction.

Paragraph 1: background on olanzapine and metabolic syndrome

“Olanzapine (OLZ) is an atypical antipsychotic drug used in the management of psychiatric disorders such as schizophrenia, depression, and bipolar disorder (1). Long-term OLZ therapy is associated with metabolic syndrome as a side effect (2,3), a complex of metabolic dysregulations including central obesity, dyslipidemia, hyperglycemia, and hypertension (3). Metabolic syndrome is linked to cardiovascular disease, type 2 diabetes mellitus, and overall increased mortality (4). However, the pathophysiologic mechanisms underlying olanzapine-induced metabolic dysregulation remain unclear. Proposed contributing factors include the disruption of appetite-regulating centers, reduced peripheral insulin sensitivity, and altered lipid metabolism (5).”

Paragrapg 2: Oxytocin’s role in metabolism

“Beyond its well-established role in reproduction, oxytocin has been found to influence feeding behavior and energy metabolism, with regard to glucose and lipid metabolism (6). Oxytocin receptors (OXTR) are present in several organs involved in energy metabolism and utilization, e.g., adipose tissue, pancreas, liver, skeletal muscle, and the hypothalamus (7). A deficiency in oxytocin signaling is associated with insulin resistance, weight gain, and dyslipidemia (8). Unlike other known intervention methods like metformin, which mainly improve insulin sensitivity and decrease hepatic glucose production(9), oxytocin has been shown to regulate metabolism through both central mechanisms, such as appetite and energy balance, and peripheral actions in metabolic organs (7). However, its involvement in olanzapine-induced metabolic syndrome is yet to be fully elucidated.”

Paragraph 3: Study objectives with a clear hypothesis

“This study aimed to explore the involvement of the oxytocinergic system as a possible pathophysiologic mechanism underlying OLZ-induced metabolic syndrome in a rodent model by, analyzing dose-dependent effects (OLZ), characterizing its biochemical features, and investigating OT’s therapeutic potential. We hypothesized that OLZ-induced metabolic syndrome is partially mediated by hypofunction of the oxytocinergic system.”

8. **Editor:** The discussion is repetitive and includes excessive mechanistic detail. Shorten it, focusing on key findings, and add a subsection addressing study limitations (e.g., use of female rats, short-term treatment, animal model constraints). Strengthen clinical implications to highlight the study’s translational potential.

**Authors’ response:** We thank the editor for this constructive feedback. The Discussion has been revised to reduce repetition and excessive mechanistic detail. We have shortened it, with greater emphasis placed on the key findings. We have also added a dedicated subsection on study limitations. We have strengthened the clinical implications to highlight the study’s translational potential.

9. **Editor:** The manuscript contains numerous typographical and grammatical errors (e.g., “titd” instead of “titled,” “mctabolic syndapars” instead of “metabolic syndrome”). Engage a professional editor or use tools like Grammarly to correct these errors.

**Authors; response:** We thank the editor for highlighting these issues. The manuscript has been carefully reviewed and corrected for typographical and grammatical errors throughout. We have used both manual proofreading and grammar-checking tools to ensure accuracy and readability.

10. **Editor:** Replace vague terms (e.g., “notable variations”) with precise scientific language (e.g., “statistically significant differences”) and break long sentences into shorter, clearer ones to improve readability.

**Authors’ response:** We have revised the manuscript to replace vague terms with precise scientific language. In addition, we have broken down long sentences into shorter, clearer ones to improve readability.

11. **Editor: References:** Update references to prioritize recent studies (2015–2025) and ensure completeness in Vancouver style

**Authors’ response:** The authors’ thank the editor for this suggestion.We have revised the reference list to prioritize recent literature (2015–2025) and to ensure full compliance with Vancouver style. Only a very small number of older citations were retained, as they are important guideline or background sources needed for context, for example, to justify doses used in the methodology and to support standard experimental procedures.

12. **Editor: Methodological Details:** Clarify group allocations in the treatment phase, justify chosen doses (e.g., oxytocin, metformin), and provide detailed protocols for assays (e.g., Butler and Mailing for hepatic triglycerides). Specify statistical methods, including software and correction methods.

**Authors’ response:** We appreciate the constructive comments.

The manuscript provides a detailed description of group allocations. To further enhance clarity, we have added a figure illustrating the group allocations.

The doses of oxytocin and metformin were selected based on previous literature. Oxytocin was adopted from an anti-obesity study, while metformin was derived from a study on olanzapine-induced metabolic syndrome, ensuring efficacy and relevance in this model. We have included these details in the Methods section and referenced accordingly.

Citation for the oxytocin dose:

Birech Z, Mwangi P, Sehmi P, Nyaga N. Application of Raman spectroscopy in comparative study of antiobesity influence of oxytocin and freeze‐dried extracts of Uvariodendron anisatum Verdeck ( Annonaceae ) in Sprague Dawley rats. J Raman Spectrosc. 2019 Nov 25;51.

Citation for metformin dose:

Luo C, Wang X, Huang HX, Mao XY, Zhou HH, Liu ZQ. Coadministration of metformin prevents olanzapine-induced metabolic dysfunction and regulates the gut-liver axis in rats. Psychopharmacology (Berl). 2021 Jan 1;238(1):239–48.

We have provided detailed protocols for all assays, including the Butler and Maling method for hepatic triglyceride estimation, in the Methods section. We have provided sufficient detail to ensure reproducibility, while also citing the original publication for reference.

The statistical methods have been specified in the revised manuscript, including the software used (GraphPad Prism 8.0.2) and the correction method (Tukey’s post hoc test for multiple comparisons).

13. **Editor:** **Visualizations**: Consider adding a figure (e.g., a line graph showing body weight changes over time) to enhance data clarity, as suggested in the detailed review comments.

**Authors’ response:** We appreciate the editor’s suggestion and have added 12 figures to enhance data clarity.

Reviewer #1:

1. **Reviewer:** The Introduction section is uncompleted and needs a key revise. The authors should explain more about the oxytocinergic system and its possible correlation with OLZ.

**Authors’ response:** We thank the reviewer for this valuable comment. In response, we have revised and expanded the Introduction to provide a more detailed explanation of the oxytocinergic system and its possible correlation with olanzapine.

The Introduction has been updated with the following paragraph: “Beyond its well-established role in reproduction, oxytocin has been found to influence feeding behavior and energy metabolism, with regard to glucose and lipid metabolism (6). Oxytocin receptors (OXTR) are present in several organs involved in energy metabolism and utilization, e.g., adipose tissue, pancreas, liver, skeletal muscle, and the hypothalamus (7). A deficiency in oxytocin signaling is associated with insulin resistance, weight gain, and dyslipidemia (8). Unlike other known intervention methods like metformin, which mainly improve insulin sensitivity and decrease hepatic glucose production(9), oxytocin has been shown to regulate metabolism through both central mechanisms, such as appetite and energy balance, and peripheral actions in metabolic organs (7). However, its involvement in olanzapine-induced metabolic syndrome is yet to be fully elucidated.”

2. **Reviewer:** What is the OT? Please add its full name before mentioning the OT for the first time in the text and also in the abstract section.

**Authors’ response:** We thank the reviewer for this comment. The full name, oxytocin (OT), has now been added at its first mention in both the main text and the Abstract. In addition, as suggested by Reviewer #3, a list of abbreviations has been included in the Supporting Information segment.

3. **Reviewer:** Did the Metformin hydrochloride tablets weigh was 500 grams?

**Authors’ response:** We thank the reviewer for pointing out this error. The unit was incorrectly written as grams in the manuscript. It has now been corrected to read “500 milligrams” for metformin hydrochloride tablets.

4. **Reviewer:** Which organ was chosen for blood drawing, after the rats were anesthetized?

**Authors’ response:** We thank the reviewer for this query. Blood samples were collected by cardiac puncture after the rats were anesthetized, and this has now been clarified in the Methods section: “Blood was collected, via cardiac puncture, in a red top vacutainer (no anticoagulant present).”

5. **Reviewer:** Which section of liver tissue was chosen to determine the triglycerides? did this section of liver tissue was same for all rats?

**Authors’ response:** We thank the reviewer for this important question. In line with the Butler and Mailing protocol, liver samples for triglyceride determination were obtained from the left lateral lobe for all rats, ensuring uniformity. This has now been clarified in the Methods section: “…portion of the homogenate (obtained from the left lateral lobe of each rat) was…”

6. **Reviewer:** The “O.d” in line 131 should be edited it can be “OD”

**Authors’ response:** We thank the reviewer for this correction. We term “O.d” has been corrected to “OD”.

7. **Reviewer:** All of the figures have low resolution and should be edited. Also, they have not error bar (SEM)

**Authors’ response:** We appreciate the reviewer’s feedback regarding the figures. Each figure has been regenerated at ≥300 dpi and saved in the required TIFF/EPS format to ensure clarity. We have also added error bars representing the standard error of the mean (SEM) to all graphs where applicable.

8. **Reviewer:** It is suggested to perform histological studies of liver tissues.
   **Authors’ response:** Thank you for this valuable suggestion. Histological studies of liver tissues were performed and are now included in the Results section (see Fig. 11).
9. **Reviewer:** The discussion section needs a major revise. The authors should discuss their results and the correlation between the results. This section should be re-wright briefly.

**Authors’ response:** We thank the reviewer for this comment. The Discussion has been revised to better integrate and correlate the key results.

Reviewer #2:

1. **Reviewer:** The method lacks strong novelty, as similar derivative blood glucose levels, oral glucose tolerance techniques have already been reported in the literature.

**Authors’ response:** We thank the reviewer for this observation. While similar techniques for assessing blood glucose and oral glucose tolerance have been described, our study goes further by evaluating additional metabolic parameters such as body weight, lipid profile and visceral adipose tissue weight, and also hepatic measurements. Moreover, we explore the effects of oxytocin on olanzapine-induced metabolic syndrome, which adds novelty to our work.

2. **Reviewer:** The abstract contains detail and should be more concise, focusing on key outcomes and significance.

**Authors’ response:** We appreciate the reviewer’s suggestion. We have revised the abstract to be more concise, focusing on key outcomes and significance.

3. **Reviewer:** The introduction provides adequate background but does not clearly justify the need for this method over existing Oxytocin, on the other hand, is known to have roles in regulation of feeding behavior, as well as lipid and glucose metabolism.

**Authors’ response:** We thank the reviewer for this insightful comment. We have revised the introduction to clearly justify the need for using oxytocin instead of existing interventions like metformin, emphasizing that while metformin mainly improves insulin sensitivity and decreases hepatic glucose production, oxytocin works through both central (appetite, energy balance) and peripheral mechanisms in metabolic organs.

We have included the following sentence to the Introduction: “Unlike other known intervention methods like metformin, which mainly improve insulin sensitivity and decrease hepatic glucose production(9), oxytocin has been shown to regulate metabolism through both central mechanisms, such as appetite and energy balance, and peripheral actions in metabolic organs.”

4. **Reviewer:** Experimental methodology is detailed, but some steps—such as wavelength selection and validation criteria—need clearer justification.

**Authors’ response:** We appreciate the reviewer’s comment. The wavelength of 540 nm was selected because it corresponds to the absorption maximum of the chromotropic acid-triglyceride complex, ensuring optimal sensitivity and specificity. Validation criteria (linearity, precision, accuracy, and limit of detection) were adopted from the original Butler and Mailing protocol, which has been widely validated in similar hepatic triglyceride analyses. We have clarified these justifications in the revised Methods section: “They were returned to the water bath for half an hour. The optical densities at 540nm, (which corresponds to the absorption maximum of the chromotropic acid-triglyceride complex), weres determined. Validation parameters such as linearity, precision, accuracy, and limit of detection, were adopted from the Butler and Mailing protocol.”

5. **Reviewer**: Figures and tables are numerous but not well integrated into the discussion, causing redundancy.

**Authors’ response:** We thank the reviewer for this helpful comment. All figures have now been carefully integrated into the Discussion. Specifically, we have discussed food intake (Figures 2 and 6), body weight (Figures 3 and 7), glycemic control [random blood glucose (Figures 4 and 8) and OGTT (Figures 5 and 9)], hepatic pathology (Figures 10 and 11), visceral adiposity (Figure 12), and dyslipidemia (Figure 13). In line with the editor’s recommendation to restructure our Results section, some figures that were originally presented together (e.g., induction and treatment data) are now shown separately to improve clarity and readability.

6. **Reviewer:** The discussion lacks comparison with alternative established methods, particularly in terms of sensitivity, accuracy, and practical advantages In contrast, central OT has been shown to exert anorexigenic effects with direct oxytocinergic neural .

**Authors’ response:** We thank the reviewer for this insightful suggestion. We have now revised the Discussion to include a comparison with established interventions such as lifestyle modification and metformin. In the revised text, we highlight that while lifestyle changes and metformin improve weight and glycemic outcomes, their effectiveness is often limited by adherence issues and a focus on downstream metabolic effects. In contrast, oxytocin offers a mechanistically distinct strategy by modulating hypothalamic pathways, with potential advantages in sensitivity to early neuroendocrine changes. We also acknowledge that further studies will be required to establish its comparative accuracy and clinical practicality.

The specific paragraph included is as follows: “While lifestyle modifications remain the preferred initial intervention for managing OLZ-induced metabolic disturbances, their efficacy is often compromised by poor adherence and the drug’s direct appetite-stimulating effects (67,68). In this study, metformin, a biguanide anti-diabetic drug (69), was used as the positive control since it has previously been found to ameliorate OLZ-induced metabolic syndrome (70–72). However, OT may offer a more targeted therapeutic approach due to its central actions compared to both lifestyle changes and metformin. Emerging evidence demonstrates that OT directly modulates hypothalamic pathways that regulate energy homeostasis and reward centers, thereby reducing OLZ-induced metabolic dysfunction in animal models (66). This central mechanism suggests potential advantages in preventing early neuroendocrine changes, unlike metformin, which mainly acts on peripheral insulin sensitization (67). Although further research is needed to confirm its relative accuracy and clinical practicality, our findings highlight its potential as a mechanistically distinct and possibly complementary strategy.”

7. **Reviewer:** The greenness evaluation (NPY/AgRP) is a strong point but lacks a deep critical analysis and clear benchmarking against literature.

**Authors’ response:** We thank the reviewer for highlighting this important point. In the revised Discussion, we have expanded the section on NPY/AgRP signaling to provide deeper critical analysis and clearer benchmarking against relevant literature. Specifically, we now highlight the central role of the H1R–AMPK–NPY/AgRP axis in olanzapine-induced hyperphagia, contextualize NPY/AgRP neurons as key integrators of energy homeostasis, and compare our findings with recent experimental evidence.

We have included the following sentences in the discussion: “However, the most consistently observed pathway involves the histamine H1 receptor (H1R) and ghrelin receptor (GHSR1a). Disruption of the interaction between H1R and GHSR1a in hypothalamic neurons, with downstream activation of adenosine monophosphate activated protein kinase (AMPK) and neuropeptide Y (NPY)/ agouti-related peptide (AgRP) signaling, has been associated with olanzapine-induced hyperphagia (22). Further, OLZ has been found to activate hypothalamic AMPK and elevate NPY expression via H1R and 5-HT2C receptor antagonism (23). Emerging research further supports the central role of AgRP neurons in integrating metabolic cues to maintain energy balance (24). Consequently, activation of NPY/AgRP neurons is recognized as a key driver of feeding behavior, strongly promoting food intake.(25).”

8. **Reviewer:** Language and grammar issues are frequent; the manuscript needs professional editing for clarity and consistency.

**Authors’ response:** We thank the reviewer for this observation. The manuscript has been thoroughly revised for grammar, language, and clarity, and we have ensured consistency throughout.

9. **Reviewer:** The conclusion is somewhat overstated and should be more balanced, acknowledging limitations and potential improvements.

**Authors’ response:** We sincerely thank the reviewer for this constructive feedback. We have revised the Conclusion to be more balanced and to better reflect the limitations already discussed. The Conclusion now acknowledges that while oxytocin attenuated olanzapine-induced metabolic dysfunction in our study, further work is required to confirm the mechanisms, establish translational relevance, and evaluate potential therapeutic applications. Below is the revised Conclusion in the manuscript:

“In this study, OT significantly attenuated olanzapine-induced metabolic dysfunction. These findings suggest that oxytocinergic hypofunction may contribute to the pathophysiology of OLZ-induced metabolic disturbances, and that modulation of this system could represent a promising therapeutic strategy. However, these results are based on preclinical models, further research is needed to confirm the mechanisms, assess translational relevance and their potential use in clinical settings.”

10. **Reviewer:** The references include some outdated or low-impact sources and suffer from inconsistent formatting and integration into the main text.

**Authors’ response**: We thank the reviewer for this valuable comment. The reference list has been thoroughly revised to prioritize recent and higher-impact sources, while retaining only a few older references that are essential for context.

Reviewer #3:

1. **Reviewer:**

Major Strengths
1. Important Topic
Olanzapine is widely used, but its side effects are a real concern. This study tackles a serious problem and explores a possible solution.
2. Clear Design
The groups and treatments are well organized. The study covers many important health markers like body weight, blood sugar, and liver fat.
3. Good Comparisons
Including a metformin group makes the study stronger, since metformin is already known to help with metabolic issues.
4. Logical Discussion
The authors give reasonable explanations for how oxytocin might work in the brain and body to reduce these side effects.

**Authors’ response:** We sincerely appreciate the reviewer’s positive assessment of our work. The encouraging feedback is highly motivating and we thank the reviewer for acknowledging these strengths.

2. **Reviewer:** No Lab Tests for Molecules
   The study talks a lot about how oxytocin and olanzapine affect certain pathways in the body. But the absence of molecular analyses (e.g., Western blot for AMPK, SREBP, GLUT4, OXTR expression) limits mechanistic conclusions. The authors should mention this as a limitation.

**Authors’ response:** We thank the reviewer for this important comment. We agree that the absence of molecular assays limits the mechanistic conclusions of our study. We have now acknowledged this as a limitation in the Discussion section and highlighted that future studies incorporating such analyses are needed to clarify the underlying signaling pathways.

3. **Reviewer**: Sex-Specific Limitations
   Using only female rats limits the study. The authors say why they chose them, but they should also clearly say that results might differ in males or humans.
   **Authors’ response:** We appreciate the reviewer for this comment. The rationale for using only female rats is detailed in the Methods section (under “Experimental animals’ selection, grouping, and treatment”), where we mention that female rats are more prone to olanzapine-induced metabolic changes based on previous studies. We agree that this choice limits the generalizability, as responses in male rats or humans may vary. We have clarified this limitation in the Discussion and recommended that future studies include both sexes to confirm the findings.
4. **Reviewer:** Oxytocin Dosage Info Missing
   The paper doesn’t explain how long oxytocin stays in the body after injection or how much actually reaches the brain. This should be briefly mentioned.

**Authors’ response:** We thank the reviewer for this important suggestion. We did not measure oxytocin levels in plasma or brain in the present study. However, we have acknowledged this as a limitation in the Discussion and added information from previous studies indicating that oxytocin administered intraperitoneally reaches peak plasma and brain levels within approximately 30 minutes and is rapidly cleared. This contextualizes our findings and highlights the need for future studies to directly assess pharmacokinetics.

5. **Reviewer**: Figures Could Be Better
   Make sure all graphs and figure legends are clear and easy to read on their own.

**Authors’ response:** We appreciate the reviewer’s feedback. All figures have been revised to improve resolution and readability, and the figure captions and legends have been expanded to ensure they are self-explanatory.

6. **Reviewer**: Writing Style
   Some parts are too formal. A few short edits could make it easier to read.

**Authors’ response**: We thank the reviewer for this helpful suggestion. The manuscript has been revised to simplify overly formal sections.

7. **Reviewer:** Say whether food intake was measured per rat or per cage.

**Authors’ response:** We thank the reviewer for this valuable suggestion. We measured food intake per cage. This has been included in the revised manuscript in the Method section under ‘Parameters assessed: food intake’.

8. **Reviewer:** Use the same units throughout the paper (e.g., mmol/L or mg/dL—don’t switch between them).

**Authors’ response:** We appreciate the reviewers for this suggestion. The units used in our manuscript reflect the standard conventions for each type of measurement. Random blood glucose and serum lipid profile are expressed in mmol/L because they are measured in fluid. On the other hand, hepatic triglycerides are expressed as expressed as mg/g to normalize to tissue weight rather than fluid volume. We have, however, ensured that within each measurement type, units are used consistently throughout the manuscript: food intake, liver weight, visceral adipose tissue weight and body weight in grams, oral glucose tolerance in mmol/L.min

9. **Reviewer:** Add a short list of abbreviations.

**Authors’ response:** Thank you for this suggestion. We have added a short list of abbreviations in the Supporting Information section in accordance with the PLOS ONE guidelines (S1 File).
